# Influence of agroclimatic factors on the efficiency of multi-ovulation in cattle in the Peruvian tropics

**DOI:** 10.3389/fvets.2025.1565265

**Published:** 2025-04-01

**Authors:** Gleni Tatiana Segura Portocarrero, Nilton Luis Murga Valderrama, Rainer Marco Lopez Lapa, José Américo Saucedo Uriarte, Deiner Jhonel Gongora Bardales, Hugo Frias Torres, Annie Yoselin Poclín Rojas, Benjamin Depaz Hizo, Ronald Will Vasquez Tarrillo, Lizeth Amparo Heredia Vilchez, Gustavo Ampuero Trigoso

**Affiliations:** ^1^Programa de Doctorado en Ciencias para el Desarrollo Sustentable de la Escuela de Posgrado (EPG), de la Universidad Nacional Toribio Rodríguez de Mendoza de Amazonas (UNTRM), Chachapoyas, Peru; ^2^Laboratorio de Biotecnología Animal, Reproducción y Mejoramiento Genético (BIOLAB) del Instituto de Investigación en Ganadería y Biotecnología (IGBI) de la Facultad de Ingeniería Zootecnista, Agronegocios y Biotecnología (FIZAB) de la Universidad Nacional Toribio Rodríguez de Mendoza de Amazonas (UNTRM), Chachapoyas, Peru; ^3^Estación Experimental Agraria (EEA)-El Porvenir del Instituto Nacional de Innovación Agraria (INIA), San Martín, Peru; ^4^Facultad de Ingeniería Zootecnista, Agronegocios y Biotecnología (FIZAB) de la Universidad Nacional Toribio Rodríguez de Mendoza de Amazonas (UNTRM), Chachapoyas, Peru; ^5^Laboratorio de Biotecnología Animal del Instituto Nacional de Innovación Agraria (INIA), San Martín, Peru

**Keywords:** environmental factors, breeds, thermal stress, physiology, *Bos indicus*

## Abstract

**Introduction:**

Agroclimatic conditions are key determinants in the development of animal production and reproduction, with specific breed differences in vulnerability to environmental stress. This research aims to determine the influence of agroclimatic factors on the efficiency of multi-ovulation in cattle in the Peruvian tropics.

**Methods:**

The study was conducted at the “El Porvenir” Agricultural Experimental Station (EEA) of the National Institute of Agricultural Innovation (INIA), located in the district of Juan Guerra, province and department of San Martín, Peru. Throughout a year, four collections of structures were made from 12 *Bos indicus* donor cows from the genetic nucleus of the PROMEG Tropical project every 2 months under intensive breeding conditions. The cows were classified according to their production: milk (five individuals of the Gyr breed and two of the Guzerat breed) and meat (two individuals of the Nelore breed and three of the Brahman breed), with ages of 3 and 4 years, selected based on specific criteria: regular estrous cycles, no deformities or reproductive problems, and certified pedigree registration. During each collection protocol, the number of viable structures (blastocysts and morulas), non-viable structures (unfertilized oocytes-UFO and degenerated), and agroclimatic factors [temperature (°C), relative humidity (%), precipitation (mm), wind speed (m/s), and the Temperature-Humidity Index (THI)] were evaluated at three times (6 a.m., 1 p.m., and 6 p.m.). A longitudinal experimental design was used for the analysis. Statistical tests were applied, including ANOVA and post-hoc tests (Tukey's Test), to assess the significance of differences between variables, such as the humidity index and temperature in relation to the production of viable structures and non-viable structures. Data visualization was achieved using R Studio libraries, including ggplot2, factoextra, and FactoMineR.

**Results:**

The analyses highlight the influence of the interaction between humidity and temperature, resulting in THI on bovine stress, revealing complex interactions that primarily affect embryo production. Stress peaks, especially under adverse conditions, were observed to significantly impact animal health.

**Discussion:**

This response to stress can affect both overall well-being and productive performance. Additionally, it should be noted that this impact varies according to the adaptability and resilience of the breed. Therefore, it is suggested to continue this study, as the literature on this topic is limited, and to conduct further research to optimize the well-being and productivity of livestock.

## Graphical Abstract

Created by Lizeth Amparo Heredia Vilchez - 2025. Microsoft^®^ PowerPoint^®^ for Microsoft 365, MSO (version 2408, build 16.0.17928.20114) for 64-bit systems. 
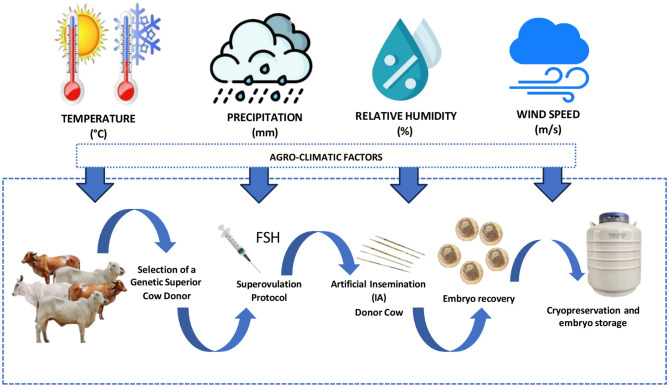

## Introduction

Located between the equator and the Tropic of Capricorn, Peru exhibits significant geographical and climatic diversity that directly influences livestock production, particularly in tropical regions where high temperatures and humidity present unique challenges (1). It exhibits remarkable geographical and climatic diversity due to the influence of the Andes mountain range and the cold ocean current. It is also characterized by a tropical climate, especially in the Amazonian plains (2). This region is distinguished by an average annual precipitation of 2,000 mm and temperatures that exceed 25°C, without a well-defined winter temperature change (3).

The jungle region is the largest in Peru, covering 60% of the national territory and characterized by a uniform tropical climate (1). This tropical climatic environment presents various challenges for livestock in the region, primarily due to high temperatures and humidity, nutrient-poor pastures, and increased susceptibility to diseases (4). This generates thermal stress in livestock and limits their ability to regulate body temperature, affecting their productive and reproductive efficiency (5). Thermal stress affects livestock's ability to regulate their body temperature, leading to a decrease in milk production, growth, and fertility (6). Vulnerability to thermal stress varies according to extrinsic factors (species, breed, production stage, size) and intrinsic factors (genetics and nutritional status of the animal) (7). For example, *Bos taurus* cattle are more susceptible to thermal stress than *Bos indicus* cattle, which are better adapted to warm climates (6).

Some of the physiological effects of thermal stress include an increased respiratory rate, decreased feed intake, dehydration, and electrolyte imbalance (8). These changes severely compromise the welfare and productivity of livestock, negatively affecting follicular dynamics, estrus detection, and the function of the fallopian tubes, leading to decreased fertility rates (9, 10).

The use of reproductive biotechnologies in livestock production has seen significant growth, as they have proven to be valuable tools for genetic improvement of livestock. These biotechnologies allow for increased embryo production, a higher number of offspring per cow, and reduced generation intervals to obtain high-quality genetic animals (11). Therefore, it is necessary to adapt and optimize advanced reproductive technologies, such as superovulation, to enhance livestock performance under the specific conditions of the Peruvian tropics. Currently, the National Institute of Agricultural Innovation (INIA) is applying different biotechnologies in livestock for tropical conditions in Peru, specifically in the El Porvenir area, where maximum temperatures reach 33°C, minimum temperatures are 19°C, and the average annual precipitation is 1,049 mm, with higher rainfall in summer and autumn (28).

The implementation of biotechnological tools has maximized the use of parental genetics, allowing for the production of a greater number of embryos and their transfer to recipient cows, enabling a high-value genetics cow to have more than ten offspring per year (12). The research and development of this adapted technology aim to evaluate the impact of agroclimatic factors on the efficiency of the superovulation technique in livestock in the Peruvian tropics.

## Methodology

### Animals and study area

The study was conducted in accordance with the guidelines established in the “Animal Protection and Welfare Law” (13). Over a period of 1 year, four collections of structures were carried out every 2 months, utilizing a selection of 12 cows from the *Bos indicus* species, classified as part of the embryo donor group, with ages ranging from 3 to 4 years. These cows were raised under intensive conditions and are certified, belonging to the national genetic core of the Instituto Nacional de Innovación Agraria. This ensures their high quality and representativeness within the country's livestock population, as part of the PROMEG Tropical project.

Additionally, it was considered that the individuals exhibited regular estrous cycles, were free from malformations and reproductive issues, and were in optimal conditions, supported by a certified pedigree record. The combination of the genetic quality of the animals and the meticulous design of the study allowed for a solid and reliable interpretation of the findings.

For each artificial insemination process, an imported straw from a bull with an outstanding pedigree was used, with a volume of 0.25 ml, sourced from Brazil (Table 1). These straws were previously evaluated using the SCA^®^ system, showing a progressive motility of 32 to 34% and a total motility of 60 to 70%. Below are the pedigrees of the bulls used:

**Table 1:** Data of bull pedigree.

**Breed**	**Bull**	**Sire**	**Dam**
Guzerat	OPIO FIV JF	PERSEUS S 5 800	NEGA TE JF
Gyr	OTTON FIV DA PALMA	C.A. SANSAO	PROFANA DE BRASILIA 17J82K/G
Brahman	CABR MUSSAMBE 2 264	JOH Wellington Manso 527/1	CABR OHIFALLA 899
Nelore	SEDUTOR COL	ONAVILLU COL | RG: COL23875	JESSA COL ^*^|RG: COLM1650(^*^)

(^*^) “Polled” or “hornless”, a result of its maternal genetics, because it is born without horns.

**Figure 1:**
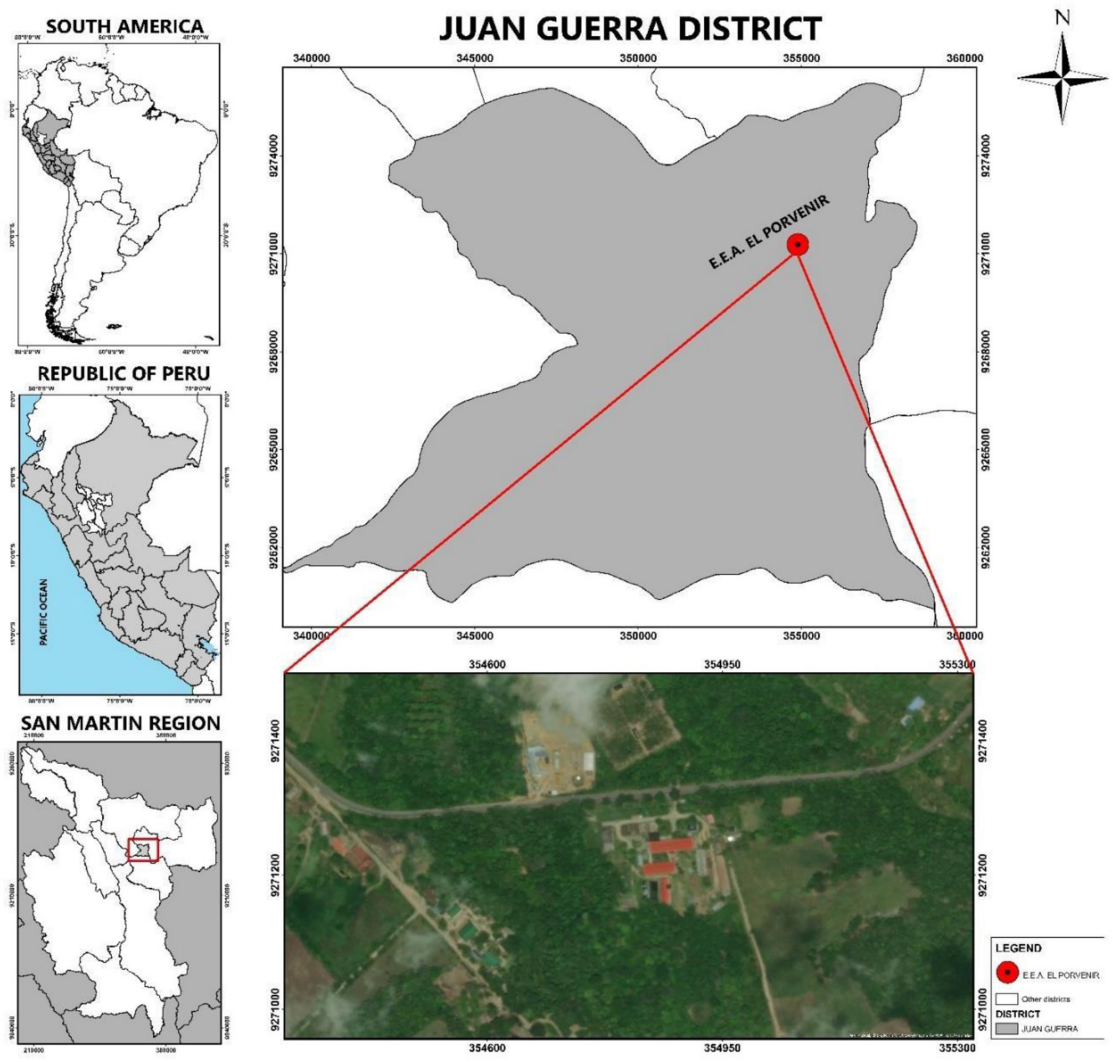
Location of the Estación Experimental Agraria (EEA) - El Porvenir of INIA (San Martin - Peru). Created by Deiner Jhonel Gongora Bardales - 2025. Esri's ArcGIS 10.5 software.

The study was conducted at the facilities of the Estación Experimental Agraria (EEA) ‘El Porvenir', belonging to the Instituto Nacional de Innovación Agraria (INIA), located in the district of Juan Guerra, province and department of San Martín, Peru (Figure 1).

### Agroclimatic factors

During each superovulation protocol, key agroclimatic parameters were recorded: temperature (°C), precipitation (mm), relative humidity (%), and wind speed (m/s) at predetermined times.

Measurements were prioritized at noon, as this is the time of day when the highest temperature levels are reached, considering it is a tropical area. This directly affects the physiological development of the animals due to the stress caused by the increase in temperature.

Climatic data were collected from the Servicio Nacional de Meteorología e Hidrología del Peru (SENAMHI) website: SENAMHI—Stations of the El Porvenir Station, located in the district of Juan Guerra, province and department of San Martín (Lat.: 6°35′20.62” S Long.: 76°19′5.66” W Alt.: 225 m above sea level). Type: Automatic-Meteorological-Code: 4723013A.

Each sampling was accompanied by the measurement of temperature and ambient humidity to calculate the temperature-humidity index (THI) in order to determine the risk of environmental thermal stress. With this data, the THI was also established, which serves as a commonly used meteorological indicator to associate the effects of heat on livestock production. This index synthesizes two of the four parameters that affect animal welfare (14) (Table 2). The THI was determined using the following formula:

**Table 2:** Monitoring thermal stress in cattle using the Temperature-Humidity Index (THI).

**RH%**	**10%**	**20%**	**30%**	**40%**	**50%**	**60%**	**70%**	**80%**	**90%**
**T**°**C**	**ITH**								
24°C	53.7	56.1	58.5	60.9	64.3	65.7	68.1	70.5	72.9
27°C	56.4	59.1	61.8	64.5	67.2	69.9	72.6	75.3	78
29°C	8.2	61.1	64	66.9	69.8	72.7	75.6	78.5	81.4
31°C	60	63.1	66.2	69.3	72.4	75.5	78.6	81.7	84.8
33°C	61.8	65.1	68.4	71.7	75	78.3	81.6	84.9	88.2
36°C	64.5	68.1	71.7	75.3	78.9	82.5	86.1	89.7	93.3
38°C	66.3	70.1	73.9	77.7	81.5	85.3	89.1	92.9	96.7
40°C	68.1	72.1	76.1	80.1	84.1	88.1	92.1	96.1	100
42°C	69.9	74.1	78.3	82.5	86.7	90.9	95.1	99.3	103.5

Legend of the Temperature-Humidity Index (THI): 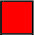 Danger of death: THI > 88.1; 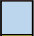 Severe stress: THI entre 78.1–88; 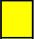 Moderate stress: THI entre 72.1–78; 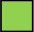 No stress experienced: THI < 72. Source (Thom, E.C., 1959).

THI = 0.8 AT° + ((HR%/100) ^*^ (AT° – 14.3)) + 46.4

Where:

- THI: Temperature-Humidity Index
- AT°: Ambient temperature (°C)
- HR%: Relative humidity

### Reproductive indicators

The following reproductive indicators were evaluated: viable structures (number of blastocysts and number of morulas) and non-viable structures (number of unfertilized oocytes—UFO and number of degenerated structures). These reproductive indicators were monitored in parallel with climatic parameters, with the aim of analyzing their behavior and possible relationships during the days when the multi-ovulation processes were carried out.

### Multi-ovulation protocol

**Figure 2:**
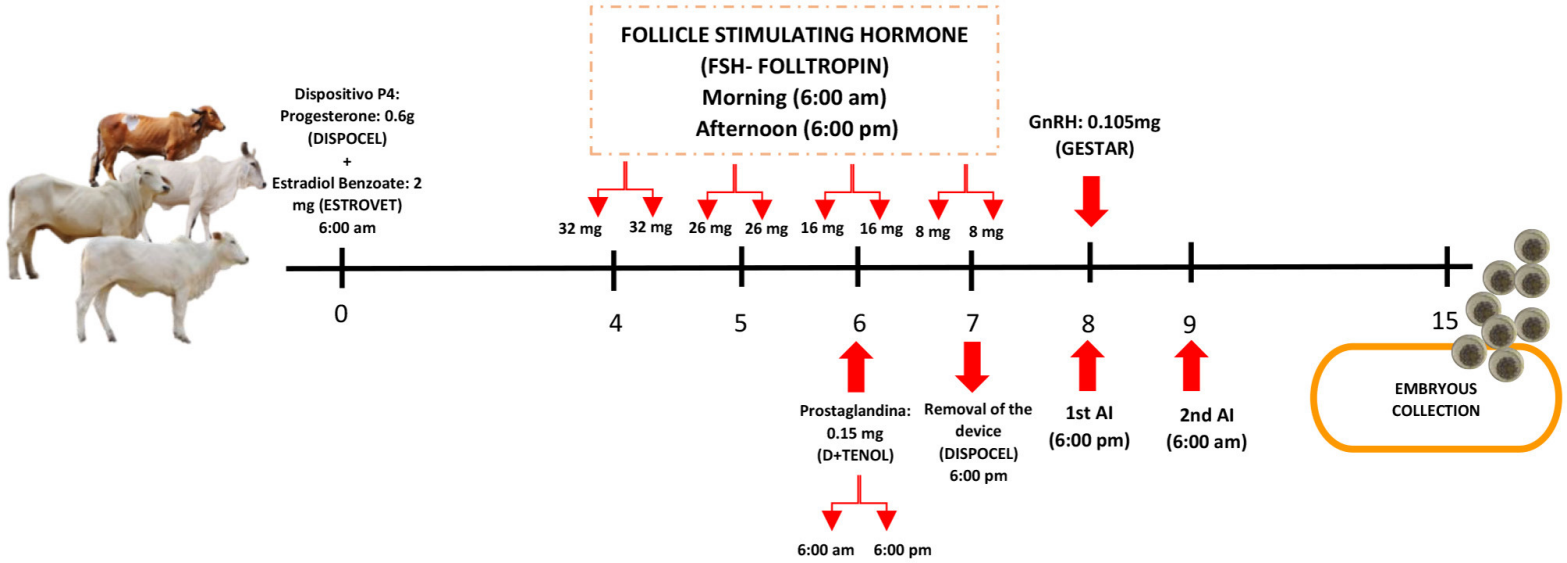
Donation protocols for the breeds: Gyr, Guzerat, Brahman, and Nelore. Created by Lizeth Amparo Heredia Vilchez - 2025. Microsoft^®^ PowerPoint^®^ for Microsoft 365, MSO (version 2408, build 16.0.17928.20114) for 64-bit systems.

In this study, a multi-ovulation protocol was implemented for *Bos indicus* cows, based on the adaptation by Baruselli et al. (15), which was applicable to both indicus and taurine breeds. This protocol was applied to the selected donor cows, received treatment protocols bi-monthly (Figure 2).

For each donor, a silicone device impregnated with 0.6 g of progesterone (P4-DISPOCEL, Von Franken, Argentina) was applied, along with 2 mg of estradiol benzoate administered intramuscularly (ESTROVET, Montana, Peru). Follicle-stimulating hormone (FSH; FOLLTROPIN^®^, Vetoquinol, USA) was administered at a total dose of 164 mg per animal, in decreasing doses over 4 days (40%-30%-20%-10%). Two doses of prostaglandin at 0.15 mg each were administered every 12 h on the third day of FSH treatment. The progesterone device was removed on the fourth day of FSH treatment, and gonadotropin-releasing hormone (GnRH) was administered in a single dose of 0.105 mg (GESTAR, Laboratorios Biogénesis Bago, Argentina) 15 h after the removal of the P4 device. The animals were inseminated 24 and 36 h after the removal of the P4 device, using a conventional straw (20 × 10^6^ spermatozoa) for artificial insemination.

### Embryo collection

**Figure 3:**
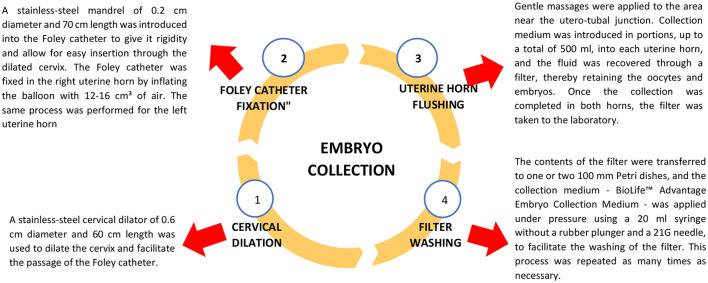
Flowchart of the embryo collection process. Created by Lizeth Amparo Heredia Vilchez - 2025. Microsoft^®^ PowerPoint^®^ for Microsoft 365, MSO (version 2408, build 16.0.17928.20114) for 64-bit systems.

The collection was performed 7 days after the first insemination (Figure 3).

### Identification and morphological evaluation

The evaluation of the collected embryos was conducted using a stereomicroscope (Nikon MZ 745) at a magnification of 20X. Additionally, the “Top View” analysis software from Nikon was used, allowing for visualization in a 2D plane and facilitating the recognition of structures. The following parameters of embryonic development were considered for this evaluation:

#### Sphericity

**Figure 4:**
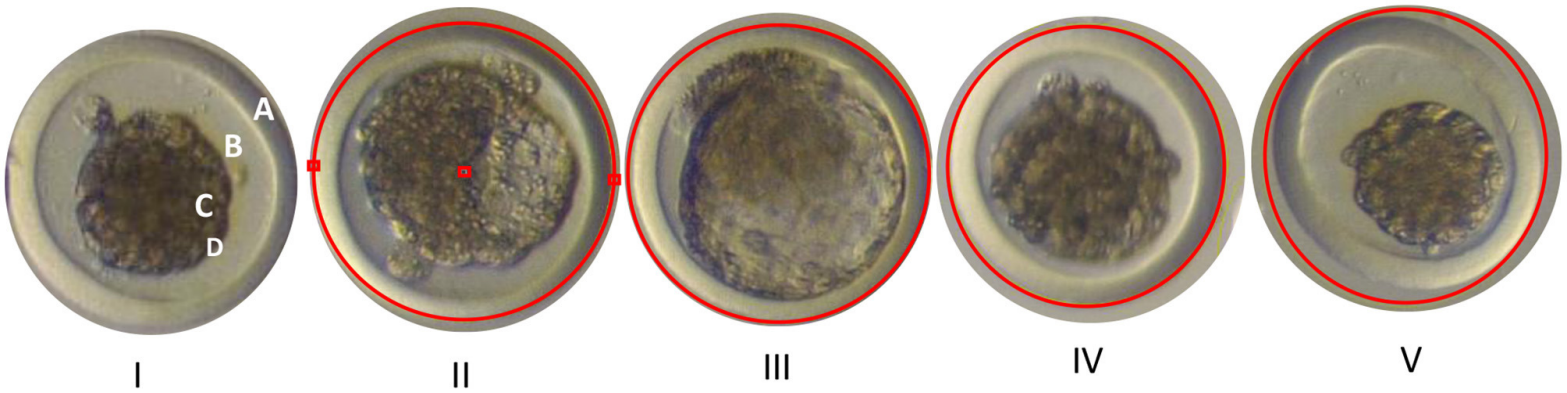
I. Structure of an embryo: **(A)** Zona pellucida. **(B)** Perivitelline space. **(C)** Inner cell mass (ICM). **(D)** Vitelline membrane. II. Reference points in the zona pellucida of the embryo. III. Expanded spherical blastocyst. IV. Specific compact morula. V. Non-spherical compact morulas. Photographs obtained with the Nikon MZ 745 stereomicroscope at a magnification of 20X, located in Laboratorio de Biotecnología Animal, Reproducción y Mejoramiento Genético (BIOLAB) from IGBI of UNTRM. Source: Own work, 2025.

The circle tool (red circle) from the “Top View” software was used, employing two parallel reference points on the zona pellucida. Embryos located within the circle were classified as spherical, while those that were not were not considered spherical (Figure 4)

#### Symmetry

**Figure 5:**
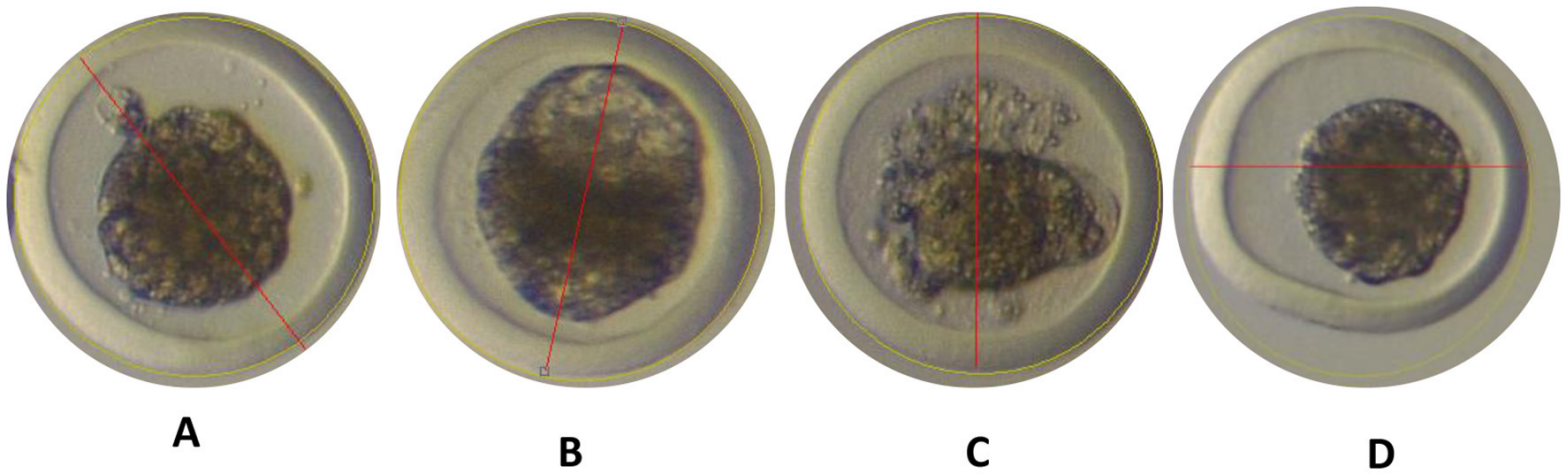
**(A, B)** Symmetrical embryos. **(C, D)** Embryos considered non-symmetrical. Photographs obtained with the Nikon MZ 745 stereomicroscope at a magnification of 20X, located in Laboratorio de Biotecnología Animal, Reproducción y Mejoramiento Genético (BIOLAB) from IGBI of UNTRM. Source: Own work, 2025.

The evaluation of symmetry was conducted by drawing a central line through the middle of the embryo, either in the “Y” or “X” plane. If the embryo displayed mirror-image characteristics, it was considered symmetrical. Symmetry was assessed across the entire embryo: cellular mass, perivitelline space, and zona pellucida (Figure 5).

#### Compact cellular mass

**Figure 6:**
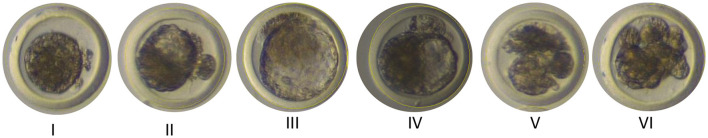
I–IV. Embryos with compact cell mass. V–VI. Non-compact cell mass. Photographs obtained with the Nikon MZ 745 stereomicroscope at a magnification of 20X, located in Laboratorio de Biotecnología Animal, Reproducción y Mejoramiento Genético (BIOLAB) from IGBI of UNTRM. Source: Own work, 2025.

The cellular mass was considered compact when at least 70% of its structure was preserved. Conversely, if more than 70% of the cellular mass was composed of extruded or degenerated blastomeres, it was classified as non-compact cellular mass. In the case of blastocysts, they were deemed compact when at least 70% of their cytological constituents (blastocoel, zona pellucida, inner cell mass, and trophoblast) were in good condition (Figure 6).

#### Cellular viability

The following classification criteria were applied, as previously described by the International Embryo Transfer Society (16):

- **Excellent Viability**: The viability of the inner cell mass is >80%. An embryo is considered excellent if its development is consistent with the collection criteria, displaying visible blastomeres, good color, uniform spherical structures with good compaction, an intact zona pellucida, and a maximum extrusion of 15%.
- **Good Viability**: The viability of the inner cell mass is >50%. This refers to an embryo with some blastomeres detached from an irregular mass, showing low compaction, intrusion into the cellular mass, cellular debris, irregular shape, low compaction, and a maximum extrusion of 50%.
- **Regular Viability**: The viability of the inner cell mass is >30%. This is characterized by a dark color (cellular debris) or a light color with irregular shapes, delayed development, a cracked zona pellucida, and a maximum extrusion of 75%.

**Figure 7:**
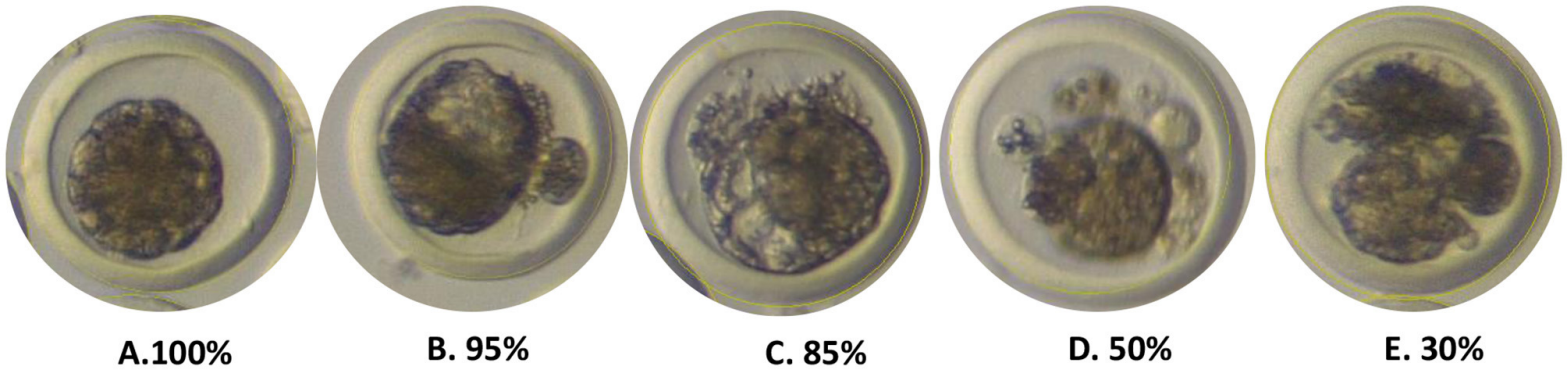
Cell viability of collected embryos. **(A–C)** Excellent viability. **(D)** Good viability. **(E)** Fair viability. Photographs obtained with the Nikon MZ 745 stereomicroscope at a magnification of 20X, located in Laboratorio de Biotecnología Animal, Reproducción y Mejoramiento Genético (BIOLAB) from IGBI of UNTRM. Source: Own work, 2025.

Considering the presence of extruded blastomeres as an indicator of cellular inviability, the percentage of the inner cell mass that was not occupied by these blastomeres was calculated. This is because extruded blastomeres tend to degenerate and do not progress adequately in embryonic development, which affects the viability of the embryo (Figure 7).

### Embryo classification

After the morphological evaluation, the embryos were transferred to a 35 mm Petri dish containing maintenance medium (Bioniche non-refrigerated Holding). Subsequently, the embryos were classified primarily based on their morphological characteristics. This comprehensive evaluation and classification process allowed for the selection of the highest quality embryos for subsequent cryopreservation.

### Data analysis

An analysis was conducted using a longitudinal experimental design. For data visualization, line and bar graphs were employed. Statistical tests, including ANOVA and *post-hoc* tests (Tukey's test), were performed to assess the significance of differences between variables, such as Temperature-Humidity Index (THI), in relation to the production of viable structures (blastocysts and morulas) and non-viable structures (unfertilized oocytes and degenerated structures).

Line graphs were used to represent the development of agro-environmental factors and the Temperature-Humidity Index, providing a clear visualization of trends and variations over time. This facilitated the analysis of the relationship between these factors and their impact on the study.

Additionally, stacked bar charts were used to visualize the production of viable structures (blastocysts and morulas) and non-viable structures (degenerated and unfertilized oocytes). This approach allows for a clear comparison of the proportion of each type of structure, facilitating the analysis of total production in a single chart while retaining most of the variability present in the variables. The libraries used for data visualization included factoextra, FactoMineR, and ggplot2. All analyses were conducted in R Studio version 4.4.2. (17).

## Results

The analysis of variance (ANOVA; Table 3) and the *post-hoc* Tukey HSD/Kramer test (Table 4) revealed significant differences in the means among the evaluated dairy breeds (Brahman and Nelore). With an F-value of 2154.21 and a *p*-value of 1.693E-20, it was demonstrated that the variability between the groups is significantly greater than within them. The group means showed that THI has an average of 77.5309, compared to the other groups (Blastocyst: 2.78125, Morulas: 2.34375, Unfertilized Oocyte: 1.375, Degenerates: 1.625). The difference in means between THI and Blastocyst was 74.7496 with a *p* < 0.0001, indicating a highly significant difference.

**Table 3:** Results of the Analysis of Variance (ANOVA)—dairy breeds.

**ANOVA**						
***Source of variation***	***SS***	***df***	***MS***	***F***	***P-value***	***F crit***
Between groups	18245.6463	4	4561.41157	2154.21357	1.693E-20	3.05556828
Within groups	31.7615553	15	2.11743702			
Total	18277.4078	19				

**Table 4:** Results of the Tukey HSD/Kramer test—dairy breeds.

Tukey HSD/Kramer		**alpha**	**0.05**	
*group*	*mean*	*n*	*ss*	*df*	*q-crit*
TUKEY HSD/KRAMER			alpha	0.05	
*group*	*mean*	*n*	*ss*	*df*	*q-crit*
Blastocyst	2.78125	4	10.2304688		
Morulas	2.34375	4	4.01171875		
Unfertilized Oocyte (UFO)	1.375	4	2.125		
Degenerates	1.625	4	4.8125		
THI	77.5308833	4	10.5818678		
		20	31.7615553	15	4.367

### Analysis of the graphs for the *Bos indicus* dairy breed

**Figure 8:**
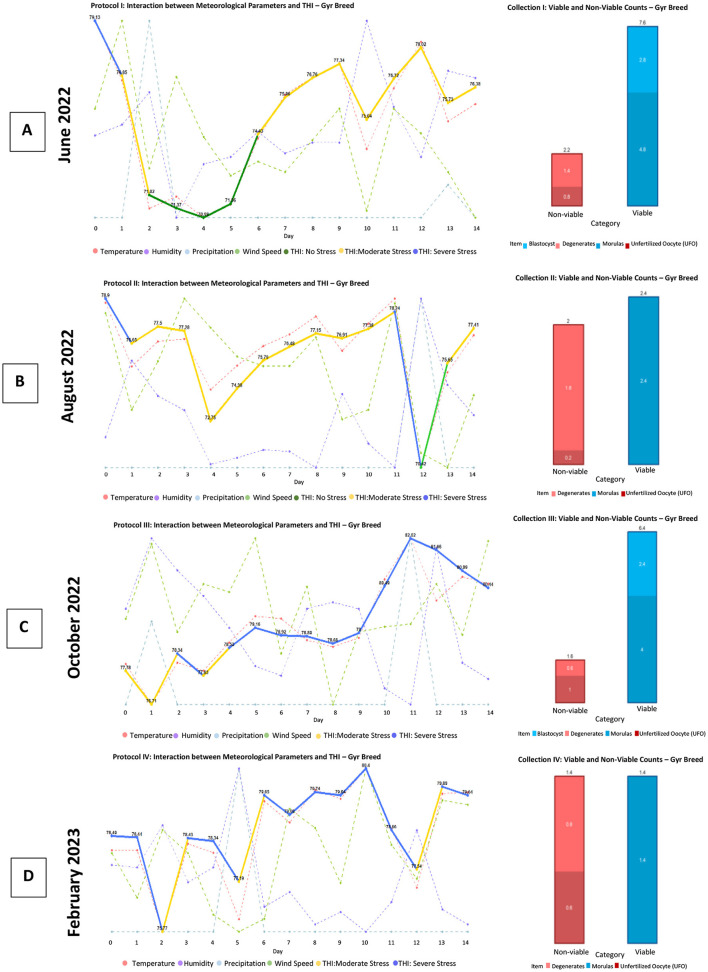
Production of viable and non-viable structures obtained with the protocol applied to the Gyr breed. This panel presents four graphs **(A–D)**, each corresponding to the fluctuation of agro-environmental factors and the Temperature-Humidity Index (THI) concerning the production of viable structures (blastocysts and morulas) and non-viable structures (unfertilized oocytes and degenerated structures) obtained at the end of each superovulation protocol for the Gyr breed. Source: Own work, 2025.

**Figure 9:**
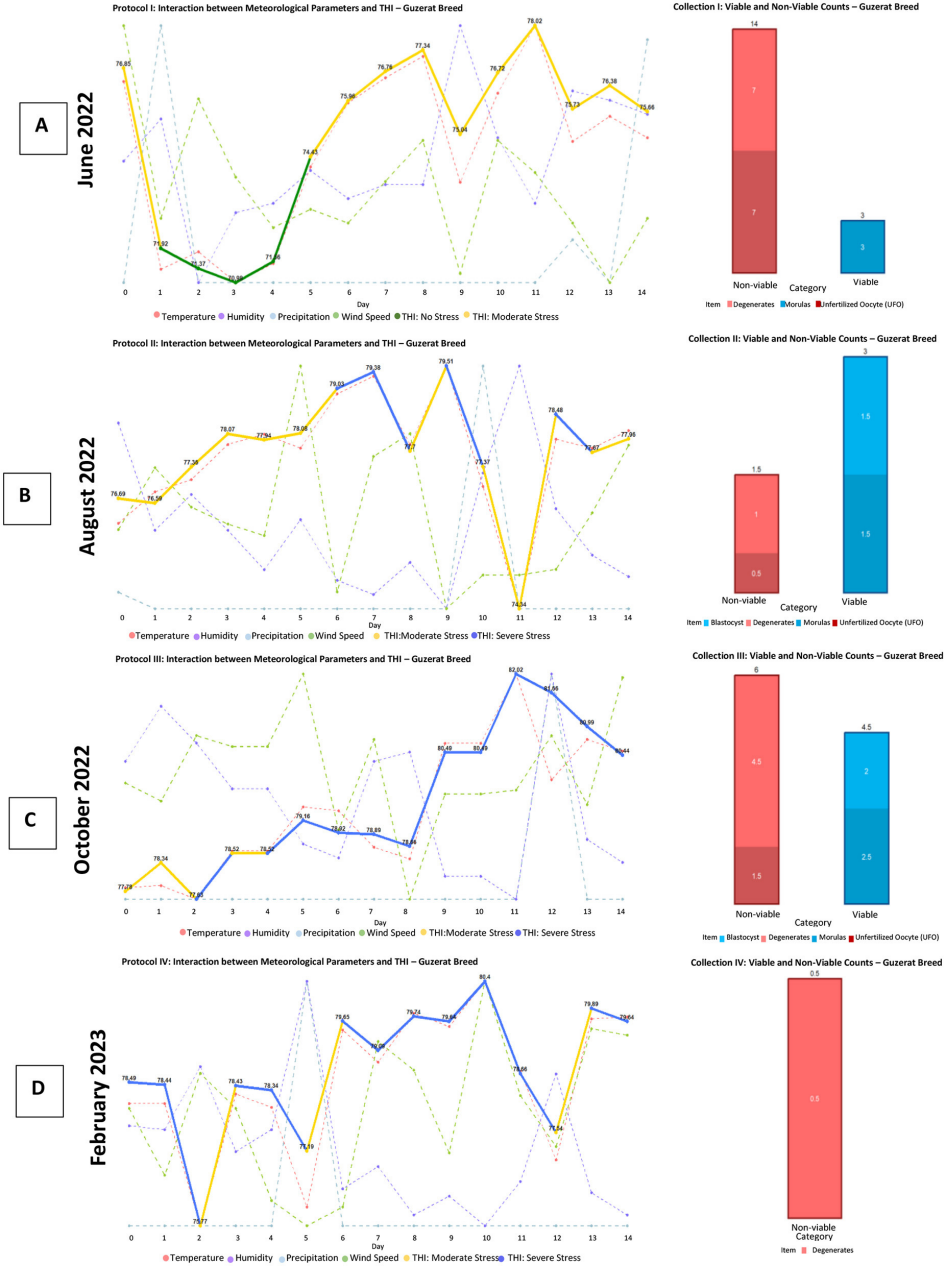
Production of viable and non-viable structures obtained with the protocol applied to the Guzerat breed. This panel presents four graphs **(A–D)**, each corresponding to the fluctuation of agro-environmental factors and the Temperature-Humidity Index (THI) concerning the production of viable structures (blastocysts and morulas) and non-viable structures (unfertilized oocytes and degenerated structures) obtained at the end of each superovulation protocol for the Guzerat breed. Source: Own work, 2025.

As seen in Figures 8B, D for the Gyr breed, as well as in Figures 9B, D for the Guzerat breed, abrupt changes in the Temperature-Humidity Index (THI) lead to a decrease in the production of viable embryos. Additionally, a notable decrease in the production of structures and embryos is observed starting from the fourth management protocol.

In Figure 9A for the Guzerat breed, a higher production of structures is noted; however, the production of viable embryos is minimal. This could be attributed to the animals being in a state of comfort at the beginning of FSH administration; however, as the protocol progressed, this comfort transformed into moderate stress, resulting in non-viable structures (degenerated and unfertilized oocytes).

In Figures 8A, C, and 9C, it can be observed that a gradual increase in THI does not affect the production of embryos.

The results of the analysis of variance (ANOVA; Table 5) indicate significant differences in the means of the evaluated meat breeds. The sum of squares between groups was 18427.5234, with an F-value of 2104.25634 and a *p*-value of 2.018E-20, demonstrating that the variability between the groups is considerably greater than the variability within them. This finding is supported by the results of the *post-hoc* Tukey HSD/Kramer test (Table 6), where notably different means were observed, such as the THI group, which has an average of 77.55599, compared to other groups like Blastocyst (0.7875) and Morulas (2.25). Comparisons between THI and Blastocyst show a mean difference of 76.76849 with a *p* < 0.0001, indicating a highly significant difference. This highlights the importance of considering these variations in future studies on the analyzed meat breeds.

**Table 5:** Results of the Analysis of Variance (ANOVA)—meat breeds.

**ANOVA**						
***Source of variation***	***SS***	***df***	***MS***	***F***	***P-value***	***F crit***
Between groups	18427.5234	4	4606.88084	2104.25634	2.018E-20	3.05556828
Within groups	32.8397312	15	2.18931541			
Total	18460.3631	19				

**Table 6:** Results of the Tukey HSD/Kramer test—meat breeds.

**Tukey HSD/Kramer**			**alpha**	**0.05**	
***group***	***mean***	***n***	***ss***	***df***	***q-crit***
Blastocyst	0.7875	4	1.041875		
Morulas	2.25	4	5.255		
Unfertilized Oocyte (UFO)	1.35	4	8.855		
Degenerates	2.35	4	7.855		
THI	77.5559907	4	9.83285621		
		20	32.8397312	15	4.367

### Analysis of the graphs for the *Bos indicus* meat breed

**Figure 10:**
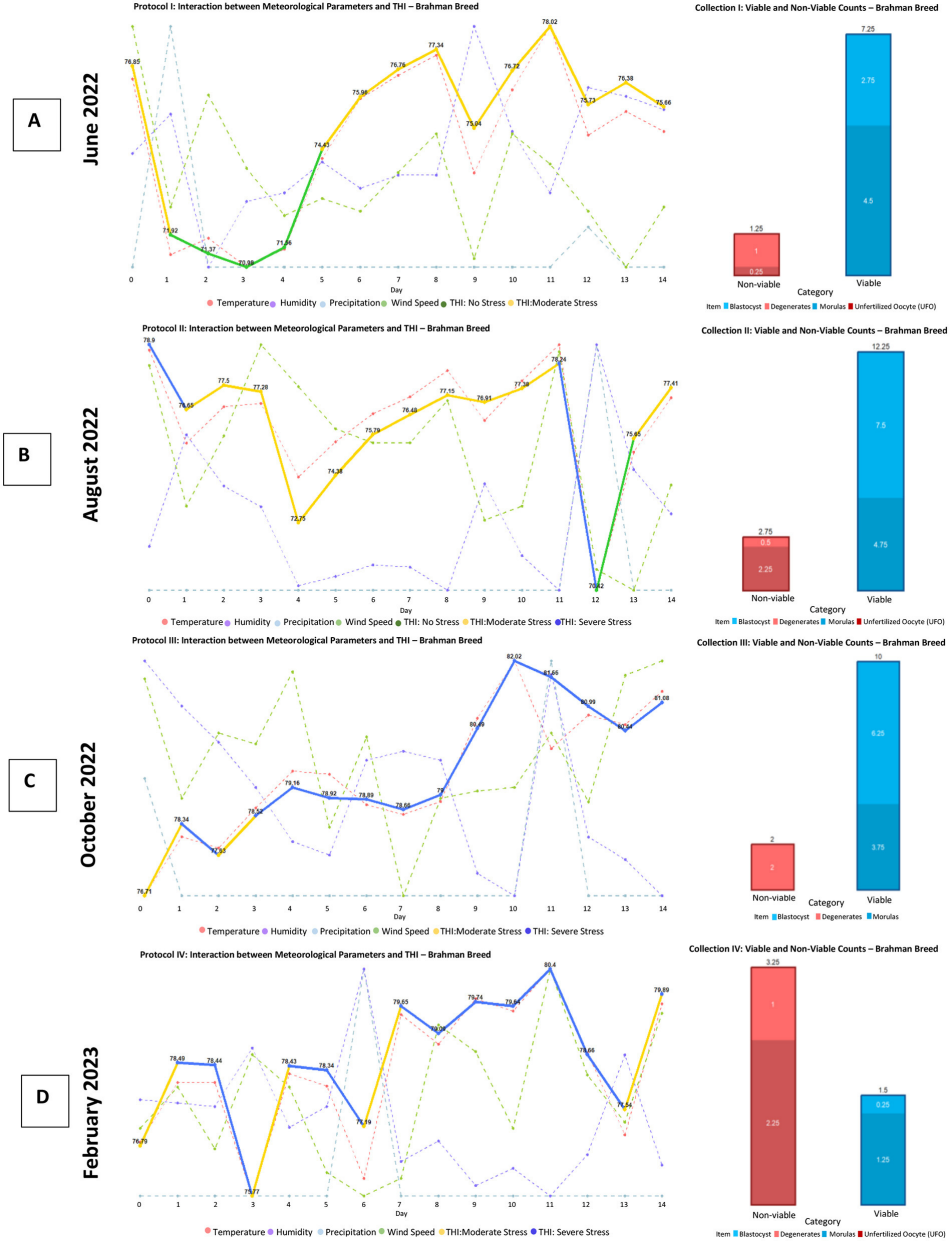
Production of viable and non-viable structures obtained with the protocol applied to the Brahman breed. This panel presents four graphs **(A–D)**, each corresponding to the fluctuation of agro-environmental factors and the Temperature-Humidity Index (THI) concerning the production of viable structures (blastocysts and morulas) and non-viable structures (unfertilized oocytes and degenerated structures) obtained at the end of each superovulation protocol for the Nelore breed. Source: Own work, 2025.

In Figure 10B, corresponding to the Brahman breed, a drastic drop in the Temperature-Humidity Index (THI) is observed. However, this decrease does not seem to affect the production of structures and embryos. This phenomenon could be explained by the animal's transition from a state of severe stress to one of comfort, which may facilitate the recovery of its reproductive capacity.

**Figure 11:**
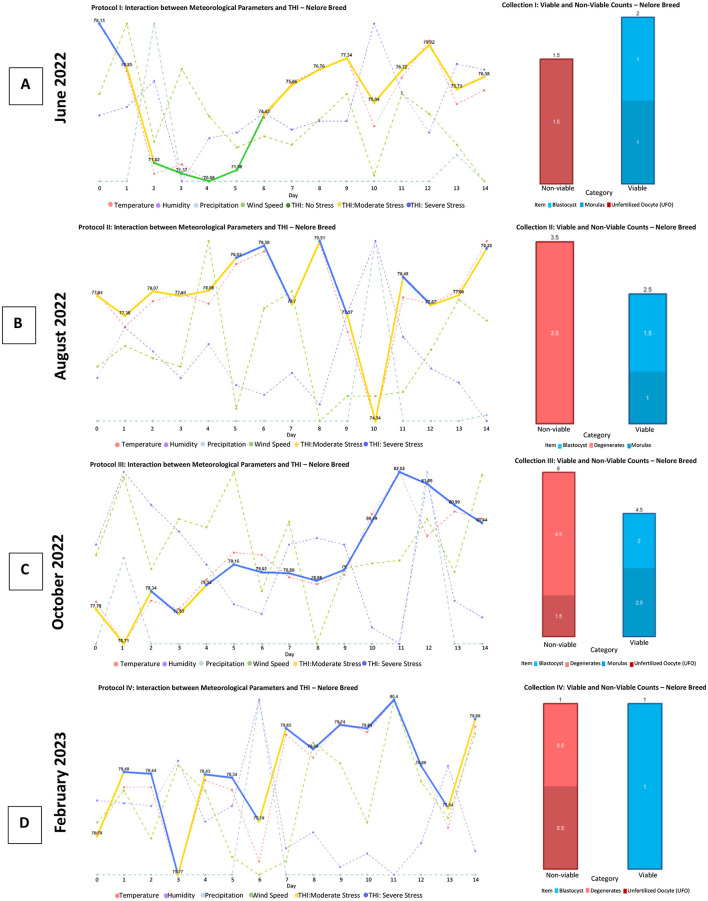
Production of viable and non-viable structures obtained with the protocol applied to the Nelore breed. This panel presents four graphs **(A–D)**, each corresponding to the fluctuation of agro-environmental factors and the Temperature-Humidity Index (THI) concerning the production of viable structures (blastocysts and morulas) and non-viable structures (unfertilized oocytes and degenerated structures) obtained at the end of each superovulation protocol for the Nelore breed. Source: Own work, 2025.

In Figure 10D (Brahman), as well as in Figures 11B, D (Nelore), it is evident that abrupt changes in the Temperature-Humidity Index (THI) lead to a decrease in the production of viable embryos. Additionally, a noticeable decline in the production of structures and embryos is observed starting from the fourth management protocol.

In Figure 10A, a THI is observed that starts with moderate stress and then declines to a condition without stress, but then rapidly fluctuates consistently at a moderate THI, resulting in a higher production of viable structures compared to non-viable structures. Therefore, it can be considered that this fluctuation favors the development of structures, but the number may be related to the application according to the protocol number. This situation is similar to Figure 10C, which in both cases could be related to having a constant THI, potentially benefiting the production of structures, although it should be noted that these ranges should be maintained at low values.

On the other hand, in Figure 11A, it can be observed that starting with severe stress directly affects the production of structures. In Figure 11C, despite also showing severe stress, there is a decrease in the number of viable structures. This can be attributed to the fact that the THI values in this situation are the highest.

When analyzing the production of viable and non-viable embryos in relation to the comfort of the animals during the multi-ovulation protocols, it is noted that a higher number of embryos were produced in the blastocyst category when the animals were in an environment with stable agroclimatic conditions, without abrupt variations.

### Average embryo production by breed

Table 7 shows the average structure values obtained by breed. For the Brahman breed, the number of embryos obtained was 7.30 ± 7.73, followed by the Gyr breed with 3.67 ± 3.86 embryos per individual, and finally, the Guzerat (2.44 ± 2.51) and Nelore (2.20 ± 1.81) breeds.

**Table 7:** Response variables in superovulation evaluated by breed.

**Estructuras**	**Indicator**	**Breed**			
		**Brahman**	**Nellore**	**Gyr**	**Guzerat**
	Embryos (N°)	7.30 ± 7.73	2.20 ± 1.81	3.67 ± 3.86	2.44 ± 2.51
Viables	Blastocysts (N°)	3.83 ± 5.65	1.30 ± 1.34	1.03 ± 2.41	0.78 ± 1.56
	Morulas (N°)	3.48 ± 4.41	0.90 ± 1.37	2.63 ± 2.59	1.56 ± 2.13
No viables	UFO (N°)	1.04 ± 1.92	0.70 ± 1.06	0.67 ± 1.21	2.00 ± 4.24
	Degenerated (N°)	0.91 ± 1.16	1.70 ± 2.06	0.93 ± 1.66	2.89 ± 3.26

Mean ± standard deviation.

It is also important to mention that the production of morulas varies by breed: Brahman (3.48 ± 4.41), Gyr (2.63 ± 2.59), Guzerat (1.56 ± 2.13), and Nelore (0.90 ± 1.37). Furthermore, the production of blastocysts varies by breed as follows: Brahman (3.83 ± 5.65), Nelore (1.30 ± 1.34), Gyr (1.03 ± 2.41), and Guzerat (0.78 ± 1.56).

### Embryonic morphology related to breed

Table 8 shows the evaluation of embryonic morphology related to breed. The Brahman breed exhibits the highest percentages of embryos with a spherical shape (60%), symmetrical structure (48%), and presence of cell mass (49%) compared to the other evaluated breeds. The Gyr breed presents intermediate results, with 31% of spherical embryos, 41% with cell mass, and 38% of symmetrical embryos.

**Table 8:** Embryonic morphology according to breed.

**Breed**	**Spherical**		**Symmetrical**		**Cellular mass**	
	**Yes**	**No**	**Yes**	**No**	**Yes**	**No**
Brahman	60%	37%	48%	48%	49%	47%
Guzerat	8%	10%	7%	10%	8%	14%
Gyr	31%	44%	41%	34%	38%	33%
Nelore	1%	10%	3%	8%	5%	6%

The Guzerat breed shows low percentages in these embryonic morphological characteristics, with only 8% of spherical embryos, 7% with cell mass, and 8% of symmetrical embryos. However, the Nelore breed records the lowest percentages across all evaluated characteristics, with only 1% of spherical embryos, 3% with cell mass, and 5% of symmetrical embryos.

Therefore, the Brahman breed appears to have the most desirable embryonic morphological profile among the tested cattle breeds, with key indicators of embryonic quality and viability, such as spherical shape, symmetry, and presence of cell mass. The other breeds exhibit less favorable results in these important embryonic characteristics.

### Cellular viability by breed

Table 9 presents a statistical description of cellular viability by breed, where an improvement is observed in the Brahman, Gyr, and Nelore breeds compared to the Guzerat breed. This suggests that the former breeds have better performance in terms of cellular viability, which is an important indicator of embryonic quality and reproductive potential.

**Table 9:** Descriptive statistics of cellular viability.

**Breed**	**Mean**	**SD**	**Min**	**Max**
**Regular**				
Brahman	50.0	0.0	50.0	50.0
Guzerat	50.0	0.0	50.0	50.0
Gyr	47.0	0.0	40.0	50.0
Nellore	0.0	0.0	0.0	0.0
**Good**				
Brahman	80.0	0.0	80.0	80.0
Guzerat	60.0	0.0	60.0	60.0
Gyr	76.0	0.1	60.0	80.0
Nellore	75.0	0.0	75.0	75.0
**Excellent**				
Brahman	96.0	0.0	85.0	100.0
Guzerat	95.0	0.1	85.0	100.0
Gyr	97.0	0.0	85.0	100.0
Nellore	96.0	0.1	85.0	100.0

SD, Standard deviation.

### Embryo classification by breed

Table 10 shows the classification of embryos based on the number of embryos collected from each breed. It is important to highlight that this classification is conducted to evaluate and select embryos in excellent, good, and regular conditions for embryo transfer or vitrification processes. This assessment is crucial to ensure the quality and viability of embryos in assisted reproduction programs.

**Table 10:** Frequency of embryos produced in different breeds according to classification.

**Breed**	**Regular**	**Good**	**Excellent**
Brahman	25%	44%	49%
Guzerat	13%	13%	8%
Gyr	63%	38%	37%
Nellore	0%	6%	6%

## Discussion

This study demonstrates that Zebu cattle, especially the meat breeds, can transition from a state of severe stress to one of comfort (with a sharp decrease in THI) without compromising their production of viable structures. This facilitates the recovery of their reproductive capacity after periods of stress, thanks to their adaptability to changing environmental conditions. This resistance to thermal stress is supported by the description of De Armas and Áraúz (18), who highlight that zebu (*Bos indicus*) have more sweat glands and a larger skin surface area than European cattle, although with lower productivity.

On the other hand, in the case of zebu dairy breeds, it was observed that during the first collection, the higher production did not correspond to an increase in viable structures. This finding suggests that, although the animals may initially be in a state of comfort, the progression of the superovulation protocol with FSH may induce moderate stress, resulting in a higher production of non-viable structures, primarily degenerated ones, and a lower production of fertilizable oocytes (UFOs).

It has been evidenced that thermal stress increases with THI, which promotes the production of degenerated embryos and altered preovulatory oocytes, affecting processes such as ovulation, fertilization, final follicle maturation, and early embryonic development. This suggests that the effect of thermal stress persists in oogenesis and gestation rates. Therefore, managing thermal stress is essential to ensuring embryonic viability and reproductive health (19–21).

Likewise, heat stress conditions decrease the intensity and duration of ovarian estrus, affecting the dynamics of follicular waves and altering the hormonal concentrations of estrogen and progesterone. Impacting various components of the ovulatory follicle, including mural granulosa cells, intrafollicular fluid, and the cumulus-oocyte complex, affecting their maturation for fertilization. In this way, it should be noted that cows exposed to chronic thermal stress exhibit reduced conception rates. Therefore, a greater number of viable structures will be evident under stable agroclimatic conditions, which highlights the importance of a controlled environment for successful reproduction (22–24).

The Brahman and Gyr breeds are the most numerous in the tropical zone, unlike the Nelore and Guzerat breeds, which are smaller populations. It is worth noting that the Brahman breed, which is one of the zebu breeds, is known for its adaptability to adverse climatic conditions and its high fertility rate. This is due to its remarkable resilience to thermal stress, which demonstrates its production of excellent viable structures, with an average of 7.3, surpassing other breeds (18, 25–27).

In this study, a higher production of viable structures in excellent condition was obtained, predominantly from the Brahman breed (49%), followed by the Gyr breed (37%), and with lower production from the Guzerat (8%) and Nelore (6%) breeds. These findings underscore the importance of the Brahman breed in the production of excellent viable structures in tropical environments, suggesting its potential to optimize livestock productivity under adverse climatic conditions.

Finally, through the obtained results, it has been demonstrated that the production of viable structures achieved through multi-ovulation protocols decreases dramatically starting from the application of the fourth protocol, limiting the effectiveness of this biotechnology. However, it is suggested to breed these animals to restore their reproductive cycle; additionally, oocyte collection (OPU) can be used as an alternative for *in vitro* embryo production, maximizing their reproductive and genetic potential, thereby contributing to the improvement of animal genetics. These findings indicate the need for management strategies that minimize environmental variations to optimize embryonic production.

## Conclusion

The findings emphasize the importance of adopting a comprehensive approach to livestock reproduction that not only focuses on quantity but also on the quality of viable structures and treatment conditions. It is essential to implement strategies that reduce stress and promote animal welfare, as these factors are key to improving embryonic viability and advancing toward a more ethical and efficient livestock system.

## Data Availability

The original contributions presented in the study are included in the article/supplementary material, further inquiries can be directed to the corresponding authors.
