# Corrigendum: Influence of agroclimatic factors on the efficiency of multi-ovulation in cattle in the Peruvian tropics

**DOI:** 10.3389/fvets.2025.1622870

**Published:** 2025-06-06

**Authors:** Gleni Tatiana Segura Portocarrero, Nilton Luis Murga Valderrama, Rainer Marco Lopez Lapa, José Américo Saucedo Uriarte, Deiner Jhonel Gongora Bardales, Hugo Frias Torres, Annie Yoselin Poclín Rojas, Benjamin Depaz Hizo, Ronald Will Vasquez Tarrillo, Lizeth Amparo Heredia Vilchez, Gustavo Ampuero Trigoso

**Affiliations:** ^1^Programa de Doctorado en Ciencias para el Desarrollo Sustentable de la Escuela de Posgrado (EPG), de la Universidad Nacional Toribio Rodríguez de Mendoza de Amazonas (UNTRM), Chachapoyas, Peru; ^2^Laboratorio de Biotecnología Animal, Reproducción y Mejoramiento Genético (BIOLAB) del Instituto de Investigación en Ganadería y Biotecnología (IGBI) de la Facultad de Ingeniería Zootecnista, Agronegocios y Biotecnología (FIZAB) de la Universidad Nacional Toribio Rodríguez de Mendoza de Amazonas (UNTRM), Chachapoyas, Peru; ^3^Estación Experimental Agraria (EEA)-El Porvenir del Instituto Nacional de Innovación Agraria (INIA), San Martín, Peru; ^4^Facultad de Ingeniería Zootecnista, Agronegocios y Biotecnología (FIZAB) de la Universidad Nacional Toribio Rodríguez de Mendoza de Amazonas (UNTRM), Chachapoyas, Peru; ^5^Laboratorio de Biotecnología Animal del Instituto Nacional de Innovación Agraria (INIA), San Martín, Peru

**Keywords:** environmental factors, breeds, thermal stress, physiology, *Bos indicus*

In the published article, there was an error in the Funding statement.

The incorrect Funding statement reads:

The author(s) declare that financial support was received for the research and/or publication of this article. This research was funded by the Programa de Doctorado en Ciencias para el Desarrollo Sustentable of the Escuela de Posgrado (EPG) at the Universidad Nacional Toribio Rodríguez de Mendoza de Amazonas (UNTRM). Additionally, it was supported by the project with unique code No. 2338934: “Improvement of the Availability and Access to Genetic Material through Reproductive Biotechnology Techniques in Tropical Livestock in the Regions of San Martín, Loreto and Ucayali PROMEG TROPICAL.” This project is associated with the Instituto Nacional de Innovación Agraria (INIA).

The correct Funding statement appears below.

